# The onset of visual experience gates auditory cortex critical periods

**DOI:** 10.1038/ncomms10416

**Published:** 2016-01-20

**Authors:** Todd M. Mowery, Vibhakar C. Kotak, Dan H. Sanes

**Affiliations:** 1 Center for Neural Science, New York University, 4 Washington Place, Room 810, New York, New York 10003, USA

## Abstract

Sensory systems influence one another during development and deprivation can lead to cross-modal plasticity. As auditory function begins before vision, we investigate the effect of manipulating visual experience during auditory cortex critical periods (CPs) by assessing the influence of early, normal and delayed eyelid opening on hearing loss-induced changes to membrane and inhibitory synaptic properties. Early eyelid opening closes the auditory cortex CPs precociously and dark rearing prevents this effect. In contrast, delayed eyelid opening extends the auditory cortex CPs by several additional days. The CP for recovery from hearing loss is also closed prematurely by early eyelid opening and extended by delayed eyelid opening. Furthermore, when coupled with transient hearing loss that animals normally fully recover from, very early visual experience leads to inhibitory deficits that persist into adulthood. Finally, we demonstrate a functional projection from the visual to auditory cortex that could mediate these effects.

## Abstract (web summary)

Visual and auditory systems influence each other during development. Here, the authors show that the onset of eyelid opening regulates critical points during which the auditory cortex is sensitive to hearing loss or the restoration of hearing

## Graphical abstract

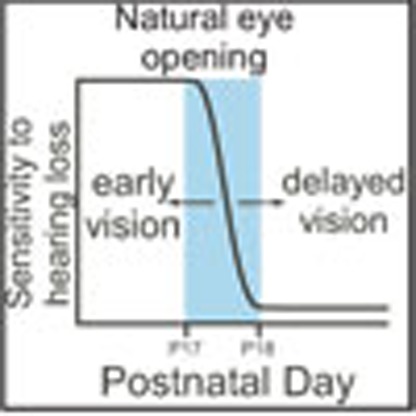

The onset of visual experience influences central auditory development. Because of this, both early and delayed visual experience can disrupt auditory processing^1^,^2^,^3^,^4^,^5^,^6^. For example, the atypical onset of auditory and visual experience in premature infants may place them at risk for maladaptive sensory processing^7^,^8^. Each sensory system displays heighted sensitivities to environmental experience called critical periods (CPs)^9^,^10^,^11^,^12^. Deprivation during these CPs can lead to the maladaptive reorganization of neuronal arbors; thereby allowing them to integrate the function of two or more intact sensory systems. This is known as cross-modal plasticity. Considering that visual experience influences the development of auditory processing, we asked whether the onset of eyelid opening regulates CPs during which the auditory cortex (ACx) is sensitive to hearing loss or the restoration of hearing.

The onset of environmental stimulus transduction displays a common sequence in vertebrates, progressing from somatosensory to vestibular, olfactory, auditory and, finally, to the visual system^13^. Thus, auditory function and auditory CPs begin before the onset of visually evoked activity^14^,^15^,^16^,^17^,^18^,^19^,^20^. For some properties, a brief window of sensitivity to auditory experience closes before eyelid opening. This window closes within days of the onset of environmental auditory stimuli transduction when ear canals open. These properties include membrane and firing properties^21^, tonotopic map formation^22^ and thalamocortical connectivity^23^. However, other properties display an extended window of sensitivity to auditory experience that overlaps with the visual CP^9^,^24^,^25^. These properties include a subset of the originally sensitive membrane and firing properties^21^, binaural integration^26^, environmental acoustic preference^27^ and spectral tuning^28^.

This window of sensitivity to hearing loss closes approximate to eye opening^21^, suggesting a correlation between the closure of the CP and the onset of visual experience. Therefore, we hypothesized that the onset of environmental visual stimuli transduction closes the auditory CPs through a functional cross-modal pathway. To test this, we experimentally manipulated eyelid opening during the CP when ACx membrane, and inhibitory synaptic properties are sensitive to hearing loss. In addition, we used anatomical tracing techniques, to demonstrate direct inputs from visual cortex (VCx) to ACx, and then developed a VCx to ACx brain slice preparation to demonstrate VCx-evoked activity in ACx. Early eyelid opening leads to a precocious termination of ACx CPs. In contrast, delayed eyelid opening leads to an extension of ACx CPs. Thus, we propose that direct functional projections from the VCx to ACx mediate these effects. These findings suggest that if the natural sequence of sensory function onset is perturbed, such as in premature infants, then the discrete CPs of developmental plasticity may be disrupted. In this way, early visual experience could lead to the persistent deficits in auditory processing reported in children born prematurely.

## Results

### Natural eyelid opening closes ACx CPs

**Figure 1:**
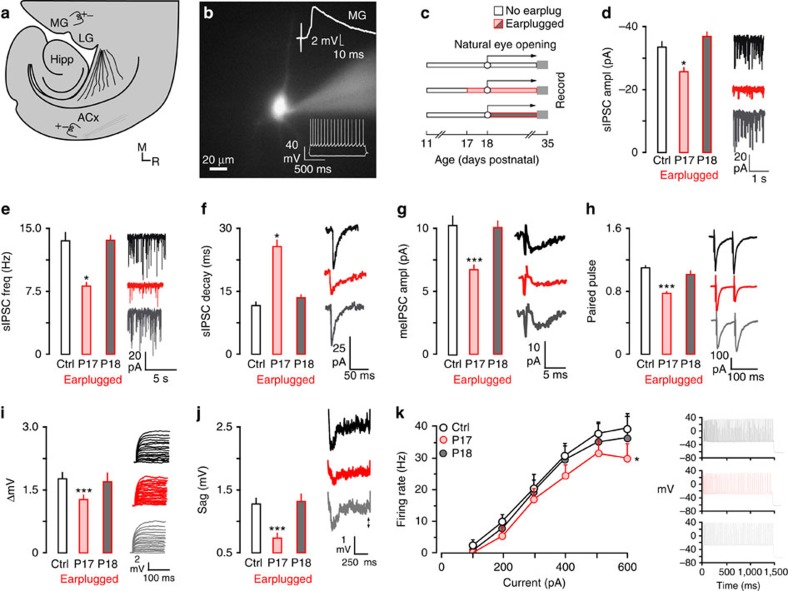
Natural eyelid opening closes the ACx CPs of sensitivity to hearing loss. (**a**) Diagram illustrates the thalamocortical brain slice preparation (ACx, auditory cortex; Hipp, hippocampus; LG, lateral geniculate; MG, medial geniculate;). The approximate position of the recording electrode in ACx and the stimulating electrodes in MG and ACx are shown. (**b**) A fluorescent image of a neurobiotin-labelled pyramidal neuron. An MG-evoked EPSP validated that neurons were located in thalamo-recipient ACx (top right). Representative neuronal responses to injection of current pulses were used to validate a regular pyramidal cell spiking pattern (bottom right). (**c**) Diagram illustrates the experimental design for data presented in **d**–**k**. Earplugs were inserted when eyes were naturally closed or when eyes were naturally opened. Earplugs were maintained until the day of recording (P29 to 35). Sample size; IPSCs: Control, *n*=40; P17, *n*=21; P18, *n*=20; Intrinsic: Control, *n*=57; P17, *n*=19; P18, *n*=24. (**d**–**f**) Bar plots (left) and traces (right) show decreased sIPSCs amplitude and frequency, as well as slower decay time constants when ears were plugged before natural eyelid opening (P17), but not after (P18). (**g**,**h**) Bar plots (left) and traces (right) show decreased meIPSC amplitude and paired pulse depression when ears were plugged on P17, but not P18. (**i**–**k**) Bar plots (left) and traces (right) show that ΔmV, sag and maximum firing rate are decreased when earplugs were inserted on P17, but not P18. Experiments were not replicated. Tukey's (honestly significant different) test, *P<0.05, **P<0.01, ***P<0.001. Error bars are s.e.m.

ACx pyramidal neurons are sensitive to transient hearing loss during developmental CPs^21^. However, we noticed that closure of the CPs correlated with natural eyelid opening. Therefore, we asked whether the onset of visual experience regulates the closure of the CP of sensitivity to hearing loss in ACx. To address this, we induced hearing loss with bilateral earplugs either before (postnatal day (P) 17) or after (P18) natural eyelid opening. Earplugs were maintained until the day of recording (P29–P35). We then determined how membrane and inhibitory synaptic properties (inhibitory postsynaptic currents (IPSCs)) differed between animals that had hearing loss induced before (*n*=13) or after (*n*=15) natural eye-opening by recording from gerbil ACx layer 2/3 pyramidal neurons (Fig. 1a,b) and comparing these changes with age-matched controls (*n*=30).

Certain IPSC properties were assessed in layer 2/3 ACx pyramidal neurons. These included spontaneous inhibitory synaptic currents (amplitude, frequency and decay time constant) and inhibitory synaptic currents evoked by layer 4 stimulation (minimum evoked (me)-IPSC amplitude, paired pulse ratios). In animals that received earplugs before natural eyelid opening, spontaneous IPSC (sIPSC) amplitudes and frequencies were significantly reduced, time constants were significantly slower, meIPSCs were significantly reduced and paired pulse ratios (PPRs) were depressed (Fig. 1d–h and Table 1). No effects were observed when earplugs were inserted after natural eyelid opening.

For membrane properties, passive (resting membrane potential (RMP)) and active properties were assessed for each layer 2/3 pyramidal neuron by injecting hyperpolarizing (input resistance (*R*_in_), decay time and sag) or depolarizing (action potential (AP) amplitude, AP threshold, AP width and ΔmV) current pulses into the cells. Only a subset of these properties was sensitive to hearing loss after P13 (see ref. 21); therefore, we expected that only ΔmV, sag and firing rate would be regulated by visual experience. Thus, we found that when earplugs were inserted before natural eye opening, ΔmV and sag were significantly smaller. Furthermore, a two-way mixed-model analysis of variance (ANOVA; control (ctrl) *n*=40, eyes closed *n*=21) showed that when the eyes were closed, maximum firing rate was also significantly reduced (eyes closed: F(1,45)=4.35, *P*<0.05), whereas all other properties were unaffected (Table 2). In contrast, no changes were seen in any properties when earplugs were inserted after natural eye opening (Fig. 1i–k and Table 2). For example, a two-way mixed-model ANOVA (ctrl *n*=40, eyes open *n*=20) showed that firing rate was not affected by hearing loss induced after natural eye opening (F(1,50)=0.05, *P*>0.1).

### Early visual experience closes ACx CPs prematurely

**Figure 2:**
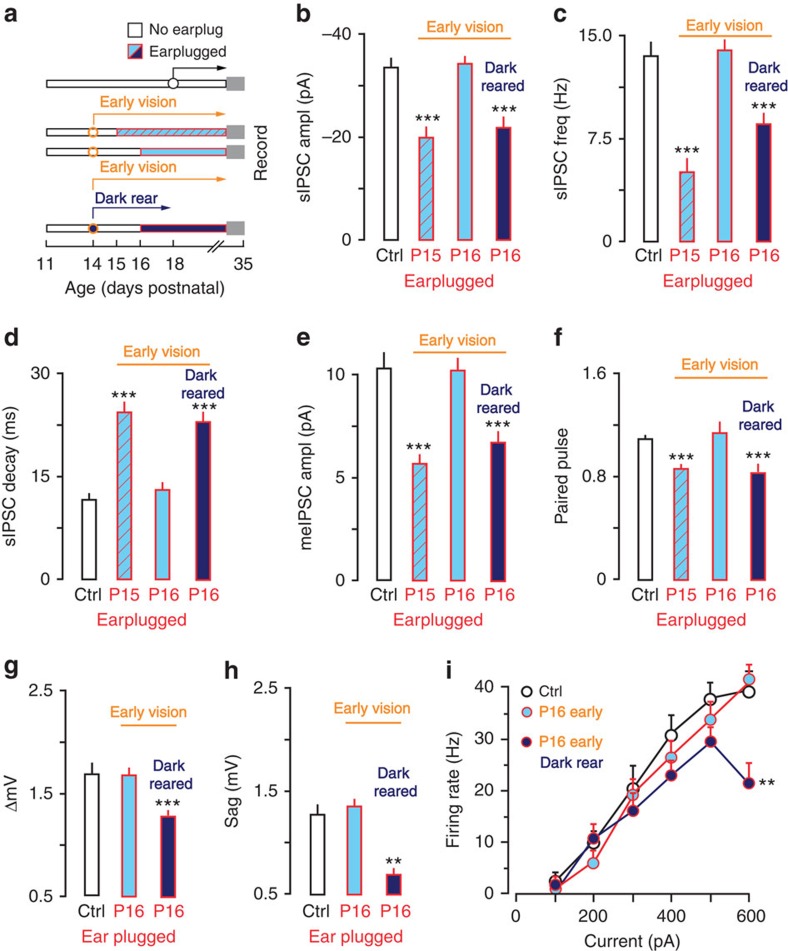
Early visual experience causes precocious closure of the ACx CPs of sensitivity to hearing loss. (**a**) Diagram shows the experimental design for data presented in **b**–**i**. Eyes were opened prematurely at P14 and ears were plugged on P15 or P16. Some animals receiving earplugs on P16 were dark reared from P14 to P17. Sample size; IPSCs: Control, *n=*40; P15, *n=*12; P16, *n=*14; P16 Dark rear, *n=*21; Intrinsic: Control, *n=*57; P16, *n=*26; P16 Dark rear, *n=*16. (**b**–**d**) Bar plots show that after early eyelid opening, sIPSC amplitude and frequency were diminished and decay time constant was slower when earplugs were inserted on P15, but not P16. Early CP closure was prevented by dark rearing. (**e**,**f**) Bar plots show that after early eyelid opening meIPSC amplitude was diminished and PPRs were depressed when ears were plugged at P15, but not P16. Dark rearing prevented early closure of the CP. (**g**–**i**) Bar plots show that after early eyelid opening the ΔmV did not decrease, depolarizing sag was not reduced and there was not a decrease in firing rate when ears were plugged on P16. Dark rearing prevented early closure of the CP. Experiments were not replicated. Tukey's (honestly significant different) test, *P<0.05, **P<0.01, ***P<0.001. Error bars are s.e.m.

To test whether premature visual experience could terminate the ACx CPs, eyelids were opened prematurely at P14 and earplugs were inserted at P15 (*n*=4) or P16 (*n*=14). Earplug insertion at P15 showed an effect of hearing loss for inhibitory synaptic properties. That is, sIPSC amplitude and frequency were reduced, sIPSC decay constant was slower, me-IPSC amplitude was reduced and PPRs were depressed. However, when eyelids were opened at P14 and earplugs were inserted at P16, both membrane properties and synaptic inhibition were no longer sensitive to hearing loss (Fig. 2b–i and Tables 1 and 2). For example, a two-way mixed-model ANOVA (ctrl *n*=40, eyes P14 EP16 *n*=14) showed that maximum firing rates were not different from controls (F(1,52)=0.02, *P*>0.1).

These results suggested that premature visual experience closed the ACx CP within 24 h. However, the surgical manipulation could have had an effect, independent of visual experience (for example, stress). To test whether visual experience closed the CP, the eyes were opened at P14 and earplugs were inserted at P16, but animals were also dark reared until the age at which eyes would naturally open (P17, *n*=12). In these animals, ACx neurons remained sensitive to hearing loss at P16. That is, sIPSC amplitudes and frequencies were reduced, time constants were slower, me-IPSCs were reduced and PPRs were depressed (Fig. 2b–f and Table 1). Similarly, ΔmV and sag were each reduced in dark-reared animals that had earplugs inserted on P16 (Fig. 2g–i and Table 2). A two-way mixed-model ANOVA (ctrl *n*=40, dark rear *n*=21) also showed that the maximum firing rate was reduced in dark-reared animals (F(1,41)=6.2, *P*<0.01). This indicated that eyelid opening terminated ACx CPs through visual experience.

### Delayed visual experience extends the ACx CP

To determine whether visual deprivation could influence the normal development of membrane and synaptic properties, animals' eyelids were glued shut until the day of recording (P25–29, *n*=13). Delayed eyelid opening retarded the natural development of some ACx membrane properties (Table 2). For example, RMP was depolarized, and AP amplitudes (AP amps) and thresholds were smaller. A two-way mixed-model ANOVA (ctrl *n*=40, delay *n*=22) also showed that maximum firing rates were reduced by visual deprivation alone (F(1, 46)=8.18, *P*<0.01). As visual deprivation significantly altered a subset of properties that are not sensitive to hearing loss after P13 (see ref. 21 and Fig. 2), we did not carry out experiments that investigated the effect of delayed visual experience on membrane property sensitivity to hearing loss.

**Figure 3:**
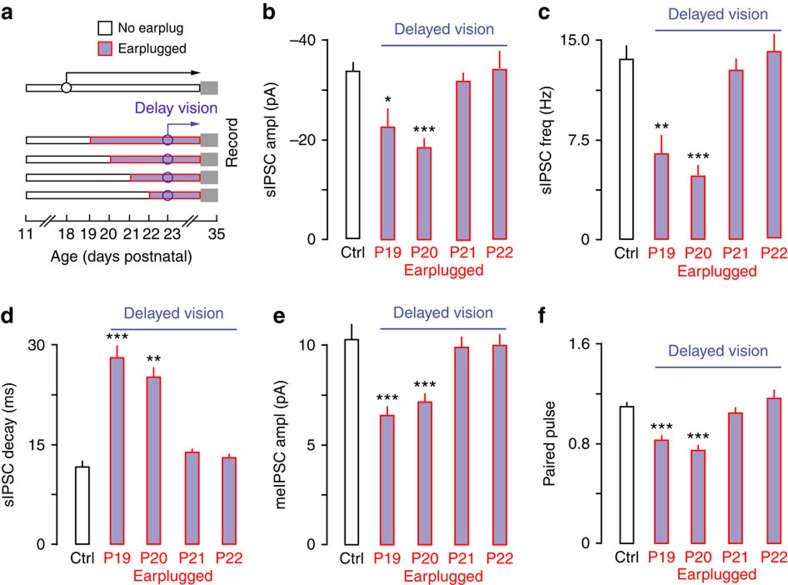
Delayed eyelid opening extends the ACx CP of sensitivity to hearing loss. (**a**) Diagram shows the experimental design for data presented in **b**–**f**. Eyes were glued shut until P23 and earplugs were inserted on P19, 20, 21 or 22 and maintained until the day of recording (P29–P35). Sample size; IPSCs: Control, *n=*40; EP19, *n=*15; EP20, *n=*14; EP21, *n=*11; EP22, *n=*22. (**b**–**d**) Bar plots show that delayed eyelid opening led to decreased sIPSC amplitude and frequency, as well as slower decay time constants when ears were plugged at P19–P20, but not P21–P22. (**e**,**f**) Bar plots show that delayed eyelid opening led to decreased meIPSC amplitude and depressed PPRs when ears were plugged at P19–P20, but not P21–P22. Experiments were not replicated. Tukey's (honestly significant different) test, *P<0.05, **P<0.01, ***P<0.001. Error bars are s.e.m.

In contrast, visual deprivation had no affect on IPSCs (Table 1). Therefore, we tested whether delayed visual experience could extend ACx IPSC CPs. Animals had both eyelids glued shut until P23 and earplugs were inserted beginning at P19, 20, 21 or 22 (*n*=18). Eyelid closure extended the IPSC sensitivity to hearing loss by several days (Fig. 3b–f and Table 1). Thus, hearing loss was able to influence IPSCs when earplugs were inserted as late as P20. However, the effect of eyelid closure was not permanent. Earplug insertion at P21 or 22 did not have an effect on IPSCs, indicating that the CP had closed. These results indicate that visual deprivation extended the CP by about 3 days.

### Vision influences the recovery from hearing loss

**Figure 4:**
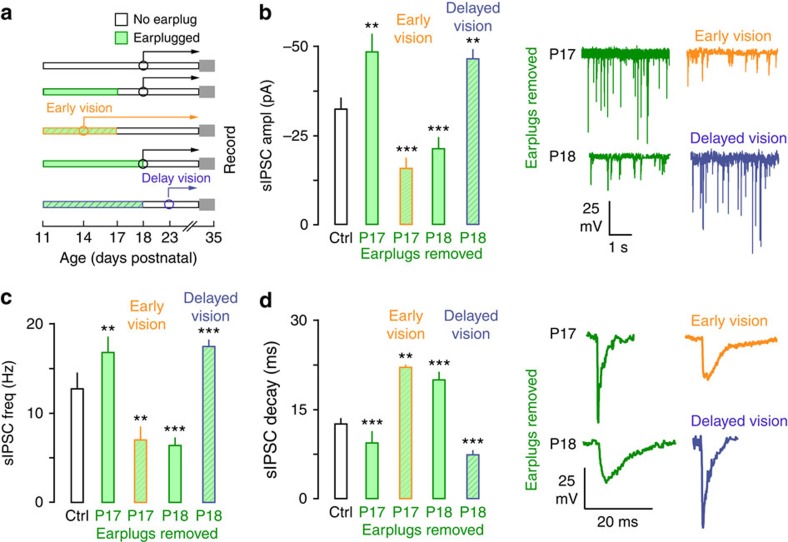
Early eyelid opening closes the ACx CP of recovery from hearing loss precociously while delayed eyelid opening extends it. (**a**) Diagram shows the experimental design for data presented in **b**–**d**. Animals experienced hearing loss from P11 to P17 or P11 to P18. One group of animals had their eyes opened at P14 and experienced hearing loss from P11 to P17. A separate group of animals had their eyes glued shut until P23 and experienced transient hearing loss from P11 to P18. Sample size; IPSCs: Control, *n=*40; Normal earplug P11 to 17, *n=*25; Normal earplug P11 to 18, *n=*15; Early earplug P11 to 17, *n=*15; Delay earplug P11 to 18, *n=*26. (**b**–**d**) Bar plots show the effect of recovery from hearing loss when eyelids were opened early (P14), opened naturally (P18) or when opening was delayed (P23). When hearing loss occurred between P11 and P17 IPSC properties were potentiated, while hearing loss that occurred between P11 and P18 lead to diminished IPSC properties. Early and delayed visual experience gated this bidirectional plasticity. That is, sIPSC amplitude and frequency were decreased and decay time constants were slower when eyes were opened prematurely (P14) and earplugs were removed at P17, indicating that the CP for recovery from hearing loss had closed early. In contrast, delayed eyelid opening (P23) led to increased sIPSC amplitude and frequency, as well as faster decay time constants when earplugs were removed at P18, indicating that the CP was extended. Experiments were not replicated. Tukey's (honestly significant different) test, *P<0.05, **P<0.01, ***P<0.001. Error bars are s.e.m.

The above experiments demonstrated that visual experience influenced the CP of sensitivity to hearing loss. We next determined whether vision also influenced the recovery of inhibition when hearing was restored (earplug removal). It was first necessary to establish the effects of restoring hearing in the absence of any manipulation to the visual system. The recovery from hearing loss was established by inserting earplugs bilaterally in animals at P11 and then removing them before (*n*=4) or after (*n*=5) natural eyelid opening (Fig. 4 and Table 3). Surprisingly, when earplugs were removed before natural eyelid opening (P17), sIPSC amplitude and frequency were greater and decay time constant was faster. No effect was observed for meIPSCs and PPRs. In contrast, earplug removal after natural eyelid opening (P18) led to smaller sIPSC amplitude and frequency, slower decay time constant, reduced meIPSCs and diminished PPRs.

Next, we tested whether visual experience regulated the CP of recovery from hearing loss. First, we assessed whether the CP closed prematurely in animals with early visual experience by coupling eyelid opening at P14 with transient hearing loss from P11 to P17 (*n*=5). We found that this caused ACx CPs to close early (Fig. 4 and Table 3). Specifically, sIPSC characteristics were similar to those observed when earplugs were removed after natural eyelid opening at P18. A second test of this concept was to determine whether the CP of recovery was extended in animals with delayed visual experience by coupling late eyelid opening at P23 with transient hearing loss from P11 to P18 (Fig. 4 and Table 3, *n*=9). We found that this manipulation extended the ACx CP. In these animals, layer 2/3 pyramidal neurons exhibited sIPSC characteristics that were similar to those observed when earplugs were removed before natural eyelid opening (P17). These findings indicate that visual experience influenced how inhibitory synaptic properties were altered following the restoration of hearing.

**Figure 5:**
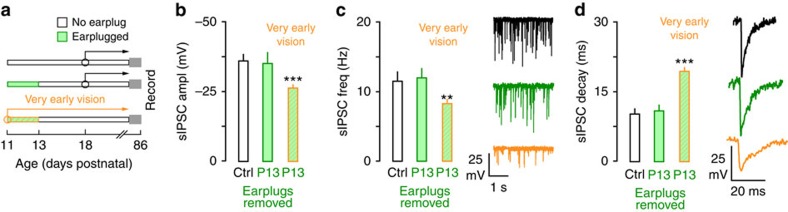
Very early eyelid opening at P11 coupled with transient hearing loss from P11 to P13 led to persistent inhibitory deficits in adults. (**a**) Diagram shows the experimental design for data presented in **b**–**d**. One group of animals received transient hearing loss from P11 to P13 and then experienced normal hearing and vision (eyes open P18) until the day of recording (P86–P92). Another group of animals experienced transient hearing loss from P11 to P13 but also had their eyes opened on P11. Animals then experienced normal hearing and vision until the day of recording (P86–P92). Sample size; IPSCs: Control, *n=*21; earplug P11 to 13, *n=*15; Early earplug P11 to 13, *n=*14. (**b**–**d**) Bar plots show complete recovery of sIPSC amplitude, frequency and decay constant in adults when earplugs were removed at P13 and eyes opened normally at P18. In contrast, sIPSC amplitude and frequency were diminished and decay time constants were slower in adults when eyes were opened very early, indicating very premature closure of the CP. Experiments were not replicated. Tukey's (honestly significant different) test, *P<0.05, **P<0.01, ***P<0.001. Error bars are s.e.m.

A final experiment was carried out to test how extremely premature visual experience affects the long-term recovery from hearing loss in adults. We first established that compared with age-matched adult controls (*n*=9) brief transient hearing loss from P11 to P13 permitted the normal development of sIPSC characteristic in adults (Fig. 5 and Table 3, *n*=5). We then tested whether very premature eyelid opening at P11 would influence inhibitory properties following recovery of transient hearing loss from P11 to P13 (*n*=6). These animals were reared to adulthood (P86) and sIPSCs were recorded. This manipulation closed the CP of recovery from hearing loss at a very early age. That is, sIPSC amplitude and frequency were smaller, decay time constants were slower, meIPSC amplitudes were reduced and PPRs were depressed (Fig. 5 and Table 3).

### Functional connectivity between visual and auditory cortices

**Figure 6:**
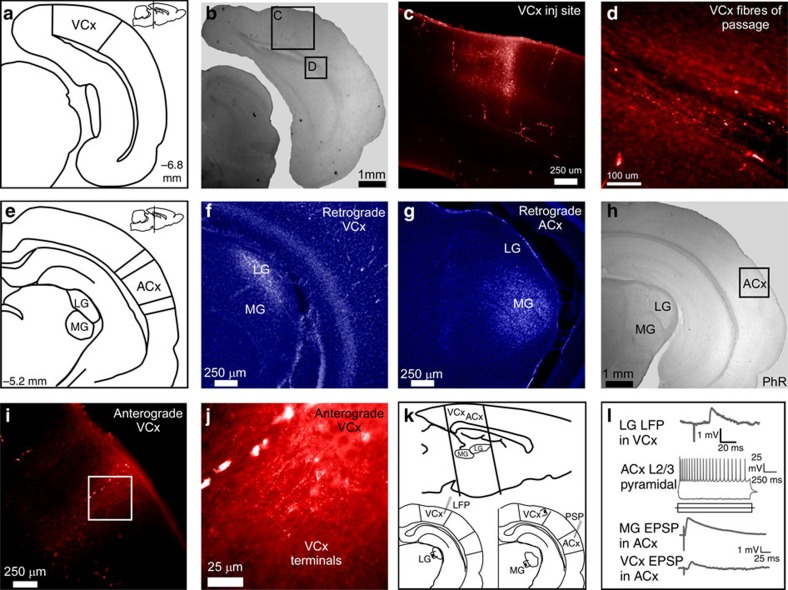
Direct functional projection from VCx to ACx. (**a**) Diagram shows the stereological location of VCx in the coronal plane and sagittal plane (insert, top right). VCx, visual cortex. (**b**) A low-magnification bright-field micrograph of a coronal slice containing the VCx injection site; scale bar, 1 mm. (**c**) VCx injection site; scale bar, 250 μm. (**d**) Fluororuby labelled fibres of passage from VCx; scale bar, 100 μm. (**e**) Diagram shows stereological locations of the LG, MG and ACx in the coronal plane and sagittal plane (insert, top right). ACx, auditory cortex; LG, lateral geniculate; MG, medial geniculate. (**f**) Fluorescent labelling of LG following injection of retrograde tracer (Fluorogold) into VCx; scale bar, 250 μm. (**g**) Fluorescent labelling of MG following injection of retrograde tracer into ACx; scale bar, 250 μm. (**h**) A low-magnification bright-field micrograph showing the general location at which anterograde labelled fibres from VCx were found; scale bar, 1 mm. (**i**,**j**) Fluorescent micrographs (× 20, × 40) showing labelled synaptic terminals in ACx layer 2/3 after injection of anterograde tracer (Fluororuby) into VCx; scale bar, 250 μm and 25 μm. (**k**) Sagittal plane diagram shows the location of the peri-coronal slice preparation used to assess functional connectivity between VCx and ACx. Local field potentials evoked by LG stimulation were used to indentify putative VCx (bottom left). Stimulating electrodes were placed in putative VCx and in MG, to evoke responses in ACx (bottom right). (**l**) An LG-evoked local field potential recorded in VCx (top). Current-evoked responses in an ACx layer 2/3 pyramidal neuron (middle). MG-evoked EPSP verified that the neuron was auditory recipient and a VCx-evoked EPSP was obtained in the same neuron (bottom).

The effects of vision on ACx CPs led us to investigate where the two systems interact. Specifically, to determine whether there are functional projections from the visual to ACx, we used neuroanatomical tracing and electrophysiological approaches in adult gerbils (Fig. 6, *n*=8). Stereological coordinates were used to inject fluorescent anterograde (fluoro-ruby) and retrograde (fluoro-gold) anatomical tracers into visual and/or ACx (see Methods). Coronal sections were then obtained and processed. We first confirmed the putative location of each cortical injection site by observing retrograde labelling in the appropriate thalamic nuclei (Fig. 6f,g). VCx injections retrogradely labelled the visual thalamus (lateral geniculate nucleus, LG) and ACx injections retrogradely labelled the auditory thalamus (medial geniculate nucleus, MG). VCx injections resulted in labelling of fibres of passage to the ACx (Fig. 6d) and terminal labelling within layer 2/3 ACx (Fig. 6h–j).

To determine whether these direct VCx projections mediated excitatory responses in ACx layer 2/3 pyramidal neurons, we developed a new brain slice preparation that preserved this pathway (see Methods). Peri-coronal sections from four animals (P17–P25) were used to assess whether auditory pyramidal neurons received functional inputs from VCx (Fig. 6k, top). A stimulating electrode was first placed in LG and evoked field potentials were used to locate approximate VCx (Fig. 6k, bottom left and Fig. 6l, top). A stimulating electrode was then placed within the VCx region that yielded an LG-evoked response and a second stimulating electrode was placed in MG (Fig. 6k, bottom right). Whole-cell current clamp recordings were obtained from ACx layer 2/3 pyramidal neurons (Fig. 6l, middle). ACx was validated by observing an MG-evoked response in the pyramidal neuron (Fig. 6l, bottom). We then demonstrated that VCx stimulation evoked excitatory post synaptic potentials (EPSPs) in ACx (Fig. 6l, bottom). Using this slice preparation we found VCx evoked synaptic responses in ACx pyramidal neurons (*n*=6) in all four animals. Together, the anatomical and functional experiments demonstrate that visual drive impinges on ACx and provides a means by which visual experience could influence auditory CPs.

## Discussion

**Figure 7:**
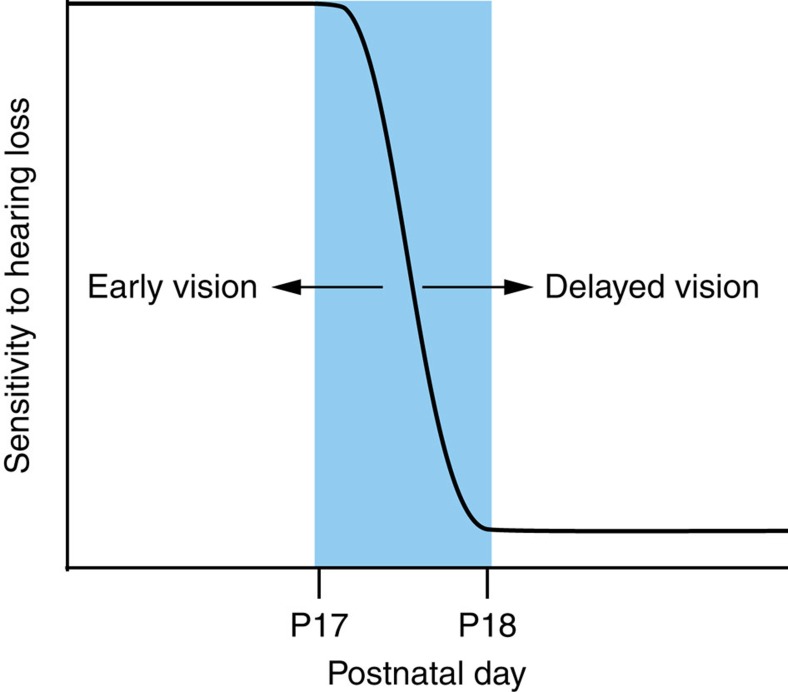
The onset of visual experience regulates ACx CPs. The schematic summarizes the effect of early, natural and late visual experience on the ACx vulnerability to hearing loss. ACx CPs close naturally between P17 and P18, when the eyelids open naturally. When eyelids are surgically opened (early vision), then the closure of ACx CPs is shifted to an earlier age range. When eyelids are glued shut (delayed vision), then the closure of ACx CPs is shifted to a later age range.

During CPs of development, the pattern of activity within a specific sensory system can induce lifelong changes for that sensory modality^9^,^10^,^11^,^12^. However, the maturation of one sensory modality depends on the precise time at which other sensory modalities begin to transduce environmental stimuli^1^,^2^,^14^,^15^. Thus, in humans blinded or deafened from birth, adaptive reorganization of neurons within deprived sensory cortices can integrate the function of two or more intact sensory systems and this is called cross-modal plasticity^8^,^29^. Here we demonstrate that the onset of visual experience regulated the CPs during which ACx membrane and inhibitory synaptic properties were sensitive to HL (Fig. 7). Specifically, the ACx CPs closed precociously by early eyelid opening and extended by delayed eyelid opening. Thus, early or late visual experience had a profound impact on ACx sensitivity to or recovery from hearing loss. There are many pathways through which visual experience could regulate ACx development, including direct activation from the visual thalamus^30^ or direct input from the VCx^31^,^32^. Here we identified direct anatomical projections from VCx to ACx and demonstrated that VCx stimulation drove EPSPs in ACx pyramidal neurons (Fig. 6). This cortico-cortical projection could provide a functional route by which developmental deprivation leads to the emergence of cross-modal plasticity.

The effects of hearing loss alone are consistent with previous sensory deprivation studies in rodents. Monocular deprivation during the first 3 days after eyelid opening leads to weaker inhibition in VCx layer 2/3 neurons^24^,^25^,^33^. Specifically, fast spiking interneuron boutons are reduced in visual and somatosensory cortices when deprivation spans all CPs^34^,^35^. In contrast, when monocular deprivation is induced during a later age, fast spiking interneuron synaptic connections onto pyramidal neurons are potentiated^35^,^36^. Our observations also revealed differential effects that depended on the relative timing of hearing loss. Thus, when a period of hearing loss began at ear canal opening (P11) and extended through P18 (when eyelids were naturally open), we observed that inhibition declined. However, when hearing loss began at P11 and ended at P17 (when eyelids were naturally closed), we observed the opposite effect: inhibition was potentiated (Fig. 4). Thus, the bidirectional effects of manipulating only the auditory system are quite similar to that observed in the visual pathway. Our results extend the principle of bidirectional inhibitory plasticity by revealing that visual experience gates such disparate effects in the ACx. Thus, early eyelid opening closed the CP of recovery prematurely, resulting in hearing loss-induced deficit to inhibitory synapses where there should have been strengthening (Fig. 4 and Table 3 early vision). In contrast, delayed eyelid opening extended the CP of recovery, resulting in hearing loss-induced strengthening of inhibitory synapses where there should have been deficits (Fig. 4 and Table 3 delayed vision). The most extreme demonstration of this principle involved opening the eyelids very early at the onset of hearing. In this case, recovery from transient hearing loss that would normally lead to full recovery instead led to a permanent reduction of IPSC properties in adults (Fig. 5).

Multisensory integration is a common feature of adult cortex^37^,^38^,^39^ and even primary sensory cortices demonstrate influences from other sensory modalities^40^,^41^,^42^,^43^,^44^,^45^. These intermodal synaptic connections are established early in development^44^,^46^, mature gradually^47^ and are highly sensitive to developmental deprivation^48^,^49^. Thus, prolonged sensory deprivation during development shifts the functional processing of the deprived sensory system towards one or more intact sensory systems^50^,^51^. That is, deprived synapses are reorganized to respond to functionally active cross-modal inputs (for example, see ref. 46). Once this occurs, the window of recovery closes and no amount of normal sensory input will lead to a remarkable improvement^52^,^53^,^54^,^55^. Our results (Figs 4 and 5) extend these principles by demonstrating that cross-modal sensory inputs can directly influence recovery from sensory deprivation by precociously terminating or extending the window of recovery. Furthermore, we observed that following visual deprivation the auditory CP was extended by many days; however, the CP did not remain open indefinitely (Fig. 3). In humans with deaf-blindness from birth, extensive somatosensory reorganization is significantly elevated in the ACx^56^. This example provides evidence that when more than one primary cross-modal input is deprived, other underlying developmental and intact sensory inputs may also regulate CPs.

When drawing comparisons between animal models and human infants, it is important to underscore that human hearing begins during gestation^57^,^58^ and visually driven activity begins postnatally^59^. Thus, relative to the normal onset of audition, infants born prematurely are exposed to visual stimuli at an abnormally early time^60^. Our experimental paradigm of early eyelid opening, which leads to the premature closure of the auditory CPs (Figs 2, 4 and 5), could provide insight into the risks associated with premature birth. Thus, our results are consistent with findings from chicks and rodents, showing that premature light exposure leads to altered auditory processing^5^,^6^,^61^,^62^. Similarly, despite normal audiometric thresholds, preterm infancy is correlated with a large range of auditory-based disorders including delayed language acquisition^63^,^64^,^65^,^66^,^67^,^68^. Therefore, we suggest that early exposure to light in preterm infants could lead to the premature closure of the auditory CP during an epoch of brain maturation vital to the development of normal auditory processing. In principle, our finding that the CP of plasticity is sustained in dark-reared animals could be used to mitigate some of the negative side effects of premature vision on auditory development in humans. In fact, preterm infants are currently being protected from extraneous sensory stimulation in the neonatal intensive care units with sensory deprivation devices (goggles and earmuffs); however, this approach may be somewhat stressful to the infant^69^. Therefore, to circumvent this problem, our results would suggest that a darkened environment could provide this benefit without stressing the infant.

## Methods

### Experimental animals

We recorded 695 pyramidal neurons from 174 male and female gerbils (*Meriones unguiculatus*), aged P11–P86 born from breeding pairs (Charles River Laboratories). Animal care and maintenance were in accordance with the guidelines and rules of the institutional care and use committee, New York University, approved by the Office of Laboratory Animal Welfare, Office of Extramural Research, U.S. National Institutes of Health. Sample size was chosen based on the number of single-cell recordings required to make reliable statistical conclusions.

### Mild hearing loss induced with bilateral earplugs

Mild hearing loss was induced by inserting a malleable plug (BlueStik Adhesive Putty, RPM International Inc.) into the opening of each ear canal. The postnatal day at which earplugs were inserted varied from P11 to P22. Detailed protocols for earplug insertion have been previously reported^21^. On the day of recording, earplugs were verified to be in place. Post-mortem examination confirmed that the tympanic membranes were intact and no cases of earplug-induced damage were observed. Earplugging increases auditory brainstem response thresholds by∼25 dB sound pressure level; however, thresholds recover rapidly after earplug removal, suggesting that the auditory periphery was not damaged^70^.

### Visual deprivation induced with bilateral eye gluing

Visual deprivation was induced by gluing the animal's eyelid on P14. Using a stereomicroscope (Olympus), the animal's head was positioned so that the eyelid was fully visible. A thin layer of super glue was then applied along the margin of each eyelid. Animals were checked once a day; however, one application was enough to hold the eyelids closed for ∼1 week. To reduce the risk of unilateral deprivation, glue was manually removed on P23 by gently tugging with blunt forceps. Eyes were inspected for damage; however, this procedure did not induce any visible damage to the sclera. No tests for the level of visual impairment were conducted.

### Premature visual experience induced by early eyelid opening

Premature visual experience was induced by manually opening the animal's eyelid on P14. The animal was gently restrained with thumb and pointer finger under a stereomicroscope (Olympus). The animal's head was positioned so that the eyelid was fully visible. Using blunt forceps the eyelid was gently pried open on both sides. Once open, the animal's eye remained open indefinitely.

### Dark rearing

To provide evidence that visual experience is required to regulate the auditory CP, animals had their eyes opened prematurely at P14 and then underwent dark rearing until P17. It is noteworthy that this duration was selected, as animals open their eyes naturally on P17. Immediately after early eyelid opening, animals were transferred to their home cage with the breeding pair. The cage was housed in a standard operant conditioning chamber that had been sealed from light exposure. The breeding pair was given *ad libitum* access to food and water, and pups continued to nurse normally. The conditioning chamber was placed in a spare room with the lights off. On P16, animals received bilateral earplugs. The conditioning chamber was moved to the ear-plugging workbench. The lights in the room remained off. Each animal was removed separately and was briefly blindfolded with fabric and surgical tape. Ears were plugged under lighting conditions that were as dim as possible, but still allowed adequate insertion of the earplug. Animals were returned to the conditioning chamber until the completion of the dark-rearing procedure. On P17, animals were returned to normal lighting environment of the animal colony and all earplugs were validated to have remained intact. Earplugs were maintained daily and all animals received normal exposure to light until the day of recording (P29–P35).

### Thalamocortical brain slice preparation

The surgery and details for thalamo-cortical brain slice preparation (500 μm) have been previously described^21^. The artificial cerebrospinal fluid contained the following (in mM): 125 NaCl, 4 KCl, 1.2 KH_2_PO_4_, 1.3 MgSO_4_, 24 NaHCO_3_, 15 glucose, 2.4 CaCl_2_ and 0.4 L-ascorbic acid, and bubbled with 95%O_2_–5%CO_2_ (pH 7.3). Before each whole-cell recording, the ACx was identified by extracellular field responses evoked by stimulation of the auditory thalamus (MG).

### Functional auditory–visual brain slice preparation

To verify that functional connectivity exists between visual cortices and auditory cortices in the gerbil, a new brain-slice preparation was developed that preserved cortical and thalamic pathways between these brain regions. First, eight gerbils were injected with retro and anterograde anatomical tracers so that projections between VCx and ACx could be identified. Animals were anaesthetized (isoflurane, 2%) and a craniotomy was made 3 mm rostral and 6.5 mm lateral to lambda over ACx^31^, and/or 6.5 mm caudal from the bregma, and 1 mm lateral from the midline over VCx. A durotomy was made over the region of interest and a pipette containing 2 μl of anterograde fluorescent tracer BDA (10% Fluoro-ruby or Fluoro-emerald, Thermo Fisher Scientific) mixed with 2 μl of retrograde fluorescent tracer BDA (10% Fluoro-gold, Fluorochrome LLC) was lowered to 0.8 mm below the pial surface. One hundred and fifty nanolitres of anatomical tracer was then injected with a Nanoject II injector (Drummond Scientific) and allowed to rest for 5 min. The pipette was retracted to 0.3 mm below the pial surface and 150 nl of anatomical tracer was again injected and allowed to rest at the cortical injection site for 5 min. The pipette was then fully retracted and the tracer was purged to verify that the pipette was not clogged. The craniotomy site covered with sterile bone wax and the surgical opening closed with Vetbond. After 1 week, animals were anaesthetized and perfused transcardially with 4% paraformaldehyde. The brains were blocked off at the cerebellum and brainstem, as well as at the level of the motor cortex. Brains were glued to the pedestal with the motor cortex facing the surface and sectioned on a vibratome (Leica) coronally at 150 μm. Sections were mounted on gelatin-coated slides, cleared with xylene and cover-slipped with VECTASHIELD mounting medium. Fluorescent/bright-field images were captured with a fluorescent camera (Zeiss AxioCam ax10, QImaging) and used to reconstruct the anatomical connectivity between VCx and ACx for slice development.

With this information a VCx–ACx brain-slice preparation was developed. Four animals (P17–P25) were deeply anaesthetized and perfused with chilled artificial cerebrospinal fluid. The brain was quickly extracted, and the cerebellum and brainstem were blocked off with a razor blade. A 10° semi-coronal cut was then made at the level of the motor cortex. The brain was placed motor cortex side down and sliced in a semi-coronal plane in the caudal to rostral direction on a vibrating microtome (Leica). After making contact with the VCx, 700 μm sections were sliced until the dorsal hippocampus and corpus callosum were clearly visible (an indicator of the end of the ACx and beginning of the somatosensory cortex). Slices were selected that visibly contained visual thalamus (LG, LG), auditory thalamus (MG), VCx and ACx.

To assess functional connectivity between VCx and ACx, a series of steps were taken for each slice (see Fig. 6k,l). First, local field potentials were used to identify regions receiving input from LG (putative VCx). A recording electrode moved medial to lateral across the dorsal regions of the slice during extracellular stimulation of the LG. Once the VCx was approximately determined, a second stimulating electrode was placed at the centre of VCx. The first biphasic stimulator was then moved to MG and another recording electrode was then used to patch layer 2/3 pyramidal neurons in the ACx. On patching, cells were assessed as healthy by the observation of RMP (< −50 mV) and the presence of overshooting APs during the injection of supra-threshold depolarizing current pulse. The cortex was then verified as thalamocortical recipient ACx by observing an EPSP in response to stimulation of MG (5 mA per 500 μS). VCx stimulation was then used to evoke synaptic responses in the ACx pyramidal neuron (*n*=6).

### Whole-cell current-clamp recordings

Current-clamp recordings (*n*=217) were obtained from pyramidal neurons in L2/3 of the ACx. Each neuron was verified as thalamo-recipient by recording an MG-evoked response and all data were collected within layer 2/3. Cells were excluded if they were found to not be thalamo recipient; however, out of 217 cells, 217 were thalamo recipient. The internal recording solution contained the following (in mM): 5 KCl, 127.5 K-gluconate, 10 HEPES, 2 MgCl_2_, 0.6 EGTA, 2 ATP, 0.3 GTP and 5 phosphocreatine (pH 7.2 with KOH). The tip resistance of the patch electrode filled with internal solution was 5–10 MΩ. Access resistance was 15–30 MΩ and was compensated by ∼70%. Passive membrane and intrinsic firing properties were evaluated on the basis of responses to positive and negative current pulses (1,500 ms). To determine AP threshold, incremental steps of current (10 pA steps) were delivered at 0.2 Hz until a spike was evoked. To investigate frequency–current curves (maximum firing rate), cells received depolarizing current injections from 100 to 600 pA (100 pA steps).

### Whole-cell voltage-clamp recordings

Voltage-clamp recordings (PC-501A; Warner Instruments, Hamden, CT) were obtained from a separate group of L2/3 pyramidal neurons (*n*=478). Each neuron was verified as thalamo recipient by recording an MG-evoked response. Cells were excluded if they were found to not be thalamo recipient; however, out of 478 cells, 478 were thalamo recipient. The internal solution contained the following (in mM): 100 KCl, 40 K-gluconate, 8 NaCl, 10 HEPES, 2 MgCl_2_, 0.1 EGTA, 2 adenosine 5′-triphosphate disodium salt, 0.3 guanosine 5′-triphosphate sodium salt and 5 Lidocaine derivative QX-314 (pH 7.2 with KOH). This internal solution contained a high chloride concentration, to obtain inward IPSCs at a holding potential of −60 mV. Briefly, we isolated sIPSC in the presence of ionotropic glutamate receptor antagonists (6,7-dinitroquinoxaline-2,3-dione 20 μM and 2-amino-5-phosphonopentanoate 50 μM). The drugs were applied for a minimum of 8 min before recording sIPSCs. At least 300 s of sIPSC data were acquired for each neuron at a holding potential of −60 mV. To examine the strength of putative monosynaptic inhibitory connections, we recorded meIPSCs at a holding potential of −60 mV. Incremental stimulus intensities were delivered to layer 4 at 0.1 Hz until an intensity was identified that produced ∼50% failures. This stimulation intensity was kept constant throughout each recording. To obtain a measure of presynaptic release, the PPRs of evoked IPSCs were used.

### Data acquisition and analysis

All data were acquired at a sampling rate of 10 kHz using a custom-designed IGOR (version 4.08; WaveMetrics, Lake Oswego, OR) macro on an iMac (Apple, Cupertino, CA). A second IGOR macro was used for offline analysis. Pyramidal neuron intrinsic membrane, and firing properties and inhibitory currents were analysed offline. For analysis, animals were assigned project numbers that blinded the analyser to experimental group. RMP, voltage threshold for AP (AP threshold), AP half-width and AP Amp, were quantified by averaging values for the first six traces after reaching AP threshold. RMP was calculated based on the 20 ms prestimulus baseline. AP threshold was calculated from the difference between the RMP and the inflection point to the first AP. AP half-width was calculated by measuring the time in milliseconds halfway between the peak of the AP and the AP threshold. AP Amp was calculated by measuring the difference in millivolts between the RMP and the AP peak. The change in membrane voltage per 10 pA step (ΔmV) was calculated by averaging the change in membrane voltage to each 10 pA step injected in the neuron before threshold. The input resistance (*R*_in_) and membrane time constant (*τ*) were quantified from traces in which a −30 pA hyperpolarizing current was injected. The sag potential was quantified by measuring the difference between the peak hyperpolarization in response to a −100 pA injected current and the subsequent asymptotic potential due to membrane depolarization. Firing rates (Hz) were quantified for each depolarizing current injection trace (100–600 pA) by counting the spikes in each trace and dividing by the duration of the current pulse (1,500 ms).

For sIPSCs, amplitudes were determined from the peak of the sIPSC to baselines continuously identified during the 30-s traces using slope thresholds. A 10-pA amplitude threshold was used to detect sIPSCs from the baseline noise. Thus, events <10 pA were not quantified. sIPSC decay time constants were measured from single exponential fits of individual sIPSCs and were excluded if a subsequent IPSC occurred within 250 ms. meIPSC amplitudes were measured from a baseline averaged for 5 ms before stimulus onset. PPRs were calculated by taking the ratio of the average IPSC amplitude evoked by the second stimulus pulse divided by the average IPSC amplitude evoked by the first stimulus pulse (IPSC2/IPSC1). If the ratio is >1 it represents paired pulse facilitation, whereas if the ratio is <1 it represents paired pulse depression. All experimentally measured values are given as mean±s.e.m. Statistical comparisons between experimental groups and age-matched controls were made with a one-way ANOVA (JMP; SAS Institute, Cary, NC). In cases where multiple group comparisons were made with a single control group, a Tukey's honestly significant different test was used as a *post-hoc* analysis to account for between-group variance and unequal sample sizes. This test yielded a *P*-value for each comparison. For *F*–*I* curves (maximum firing rate), a two-way mixed-model ANOVA was used to verify a main effect of earplugging on evoked firing rate during incremental current injection steps.

## Additional information

**How to cite this article:** Mowery, T. M. *et al.* The onset of visual experience gates auditory cortex critical periods. *Nat. Commun.* 7:10416 doi: 10.1038/ncomms10416 (2016).

## Figures and Tables

**Table 1:** Visual experience regulates the inhibitory CP for sensitivity to hearing loss.

	**sIPSC ampl. (pA)**	**sIPSC freq. (Hz)**	**sIPSC decay time (ms)**	**meIPSC ampl. (pA)**	**PPR**
Figure 1					
Ctrl (*n*=40)	−33.7±2.1	12.2±1.4	11.9±1.0	−10.4±0.8	1.1±0.04
Normal, EP17 (*n*=21)	−26.3±1.7*	8.5±0.8*	20.4±2.5*	−7.8±0.3***	0.79±0.02***
Normal, EP18 (*n*=20)	−36.6±2.0	12.5±1.0	13.8±0.7	−10.0±0.5	1.2±0.05
					
Figure 2					
Early, EP15 (*n*=12)	−20.0±1.8***	4.6±0.8***	24.5±3.0***	−6.2±0.5***	0.78±0.03***
Early, EP16 (*n*=14)	−32.3±1.9	12.9±0.7	12.8±1.2	−10.0±0.3	1.10±0.03
Early, EP16, DR (*n*=21)	−22.6±1.3***	8.3±0.6***	22.8±2.6***	−7.0±0.7***	0.84±0.04***
					
Figure 3					
Delay, EP19 (*n*=15)	−23.7±3.2*	6.15±0.8**	27.2±3.7***	−6.5±0.4***	0.84±0.02***
Delay, EP20 (*n*=14)	−18.9±1.4***	4.2±0.6***	19.7±1.7**	−6.2±0.3***	0.78±0.02***
Delay, EP21 (*n*=11)	−31.2±1.9	11.2±1.0	13.2±1.0	−9.8±0.6	1.04±0.04
Delay, EP22 (*n*=22)	−34.9±2.9	12.7±1.6	12.0±1.2	−10.0±0.3	1.19±0.07
Delay, no EP (*n*=22)	−31.1±3.2	14.5±1.2	13.9±1.2	−11.7±0.3	0.98±0.05

CP, critical period; Delay, eyes open P23; DR, dark rear 14 to 17; Early, eyes open P14; EP, earplug insertion days; meIPSC, minimum evoked inhibitory postsynaptic current; Normal, eyes open P18; PPR, paired pulse ratio; sIPSC, spontaneous inhibitory postsynaptic current.

*P<0.05, **P<0.01 and ***P<0.001.

**Table 2:** Visual experience regulates the membrane property CP for sensitivity to hearing loss.

	**RMP (mV)**	**AP Amp (mV)**	**AP thresh (mV)**	***R***_**in**_ **(MΩ)**	***τ*** **(ms)**	**AP width (ms)**	**Sag (mV)**	**ΔmV**
Figure 1								
Ctrl (*n*=57)	−64.5±0.4	97.1±1.0	−31.3±0.6	127±6.1	9.6±0.4	1.01±0.04	1.30±0.6	1.88±0.07
Normal, EP17 (*n*=19)	−64.1±0.5	95.5±1.3	−30.9±0.9	114±10.3	12.1±1.1	1.06±0.3	0.7±0.0***	1.32±0.07***
Normal, EP18 (*n*=24)	−66.3±0.8	100.0±1.9	−34.7±2.3	125±12	9.4±0.7	0.90±0.06	1.33±0. 1	1.69±0.13
								
Figure 2								
Early, EP16 (*n*=26)	−65.7±0.5	98.7±1.3	−31.6±1.1	112±10.1	10.1±0.6	0.93±0.4	1.37±0.06	1.86±0.12
Early, EP16, DR (*n*=16)	−63.0±0.4	95.3±1.0	−31.0±1.3	114±7.9	11.0±0.7	1.04±0.05	0.6±0.0***	1.28±0.07**
Delay, no EP (*n*=28)	−61.3±0.7**	90.5±1.2***	−27.6±1.4*	132±10.7	12.0±0.9	1.10±0.07	1.26±0.08	1.98±0.11

AP amp, action potential amplitude; AP threshold, voltage threshold to spike; AP width, action potential depolarizing sag; CP, critical period; DR, dark rear; Delay, eyes opened P23; Early, eyes opened P14; EP, earplug insertion days; Normal, eyes opened P17; RMP, resting membrane potential; *R*_in_, input resistance; τ, membrane time constant; ΔmV, change in membrane voltage per 10 pA step.

*P<0.05, **P<0.01 and ***P<0.001.

**Table 3:** Visual experience regulates the IPSC CP of recovery from hearing loss.

	**sIPSC ampl. (pA)**	**sIPSC freq. (Hz)**	**sIPSC decay time (ms)**	**meIPSC ampl. (pA)**	**PPR**
Figure 5					
Ctrl P35 (*n*=40)	−33.7±2.1	12.2±1.4	11.9±1.0	−10.4±0.8	1.1±0.04
Normal, EP11–17 (*n*=25)	−47.5±3.2**	16.3±0.9**	8.7±0.5***	−9.7±0.2	1.02±0.05
Normal, EP11–18 (*n*=15)	−24.6±2.4***	6.2±0.7***	18.3±1.2***	−7.9±0.3***	0.81±0.02***
Early, EP11–17 (*n*=15)	−21.3±1.9***	6.7±0.9**	20.2±3.6**	−7.3±0.5***	0.82±0.04***
Delay, EP11–18 (*n*=26)	−45.9±4.8**	17.1±0.7***	7.7±0.4***	−9.5±0.2	1.09±0.07
					
Figure 6					
Adult Ctrl, (*n*=21)	−39.4±2.1	11.8±0.9	9.5±0.6	−11.2±0.4	1.03±0.02
Adult Normal, EP11–13 (*n*=15)	−36.4±3.3	12.6±0.9	10.2±0.8	−10.7±0.8	1.00±0.06
Adult very early, EP11–13 (*n*=14)	−25.4±2.2***	8.1±0.6**	19.0±2.1***	−8.1±0.4***	0.77±0.02***

CP, critical period; Delay, eyes open P23; Early, eyes open P14; EP, earplug insertion days; meIPSC, minimum evoked inhibitory postsynaptic current; Normal, eyes open P18; PPR, paired pulse ratio; sIPSC, spontaneous inhibitory postsynaptic current; Very Early, eyes open P11.

*P<0.05, **P<0.01 and ***P<0.001.
